# Association between obstructive sleep apnea and cardiovascular events in hemodialysis patients

**DOI:** 10.1590/2175-8239-JBN-2025-0123en

**Published:** 2025-09-26

**Authors:** Ellen Goes, Skarllet Cândida Silva dos Santos, Rodrigo Bezerra, Flávio Teles

**Affiliations:** 1 Universidade Federal de Alagoas, Faculdade de Medicina, Programa de Pós-Graduação em Ciências Médicas, Maceió, AL, Brazil.; 2 Universidade Federal de Alagoas, Faculdade de Medicina, Maceió, AL, Brazil.; 3 Universidade de Pernambuco, Hospital Universitário de Pernambuco, Pronto-Socorro Cardiológico Universitário de Pernambuco, Recife, PE, Brazil.; 4 Universidade Federal de Alagoas, Faculdade de Medicina, Maceió, AL, Brazil.

**Keywords:** Renal Dialysis, Indicators of Morbidity and Mortality, Cardiovascular Diseases, Sleep Apnea, Obstructive, Sleep Quality

## Abstract

**Introduction:**

Hemodialysis patients are at high cardiovascular risk, with sudden death being one of the leading cause of mortality. Sleep disorders are highly prevalent in this population, and obstructive sleep apnea (OSA) has been associated with poorer blood pressure control and cardiovascular damage.

**Objective:**

To investigate the association between an intermediate or high risk of OSA and the occurrence of major cardiovascular events in hemodialysis patients.

**Methods:**

This prospective multicenter cohort study was conducted in three hemodialysis clinics between May 2022 and May 2024. A total of 165 patients aged 18 to 75 years who had been undergoing hemodialysis for at least 6 months were included. Clinical, labora­tory, and sleep-related variables, were assessed, including OSA risk (STOP-Bang), sleep quality (Pittsburgh Sleep Quality Index), chronotype (Morningness-Eveningness Questionnaire), and the occurrence of major adverse cardiovascular events (MACE+). Patients were followed for 22 to 24 months.

**Results:**

Overall 64.8% of patients were classified as being at an intermediate or high risk for OSA. This group showed a higher prevalence of diabetes and obesity, poorer sleep quality, more cases of chronic restless legs syndrome, and lower dialysis adequacy. The incidence of major cardiovascular events was significantly higher among patients at risk of OSA (17.0% vs. 5.2%; p = 0.03), with an independent association observed between OSA and sudden death (OR 1.18, 95% CI 1.01–1.39; p = 0.03). Other sleep disorders were not associated with increased cardiovascular risk.

**Conclusion:**

Hemodialysis patients had a high risk of OSA, which was independently associated with adverse cardiovascular outcomes.

## Introduction

Patients with chronic kidney disease (CKD) undergoing dialysis therapy are at high cardiovascular risk, with sudden death being one of the leading cause of mortality^1^. The main contributing factors include a high prevalence of diabetes mellitus, difficult-to-control arterial hypertension, obesity, dyslipidemia, mineral and bone disorders associated with vascular calcification, as well as factors such as hypervolemia and hyperkalemia^2,3^.

In this context, obstructive sleep apnea (OSA) has gained attention as a potential additional cardiovascular risk factor. OSA is associated with worse cardiovascular outcomes in the general population and, like other sleep disorders is highly prevalent in individuals undergoing hemodialysis^4,5^. Furthermore, OSA is linked to poorer blood pressure control and is more common in individuals with diabetes and obesity^6^. In patients with advanced CKD, OSA is associated with greater interdialytic weight gain, poorer blood pressure control, and lower dialysis adequacy^7, 8, 9^. However, studies investigating the correlation between OSA and cardiovascular outcomes in hemodialysis patients remain scarce^10^.

Given the multiple factors that contribute to the elevated cardiovascular risk inhemodialysis patients, the present study aimed to investigate the association between intermediate or high risk of OSA and the occurrence of adverse cardiovascular events. Additionally, the study evaluated the prevalence of other sleep disorders in this population and explored their possible correlations with clinical and cardiovascular variables.

## Methods

### Study Design

This prospective cohort study involved three data collection points: May 2022 (participant enrollment), May 2023, and May 2024. At each time point, clinical analyses and hospitalization data were collected from medical records. The study included adult patients undergoing hemodialysis at three nephrology clinics located in different regions of the state of Alagoas, in the Northeast of Brazil. The selection of geographically distributed units aimed to capture the sociodemographic diversity of the patients and ensure greater representativeness of the dialysis population in the state. This approach allowed for a more comprehensive analysis of the investigated variables, considering regional differences in social determinants and access to healthcare services^5^.

Patients aged between 18 and 75 years diagnosed with CKD and undergoing hemodialysis for at least six months with a regular frequency of three weekly sessions were included. Exclusion criteria comprised patients unable to respond to the applied questionnaires due to clinical conditions, those with irregular attendance at hemodialysis sessions, melatonin users, individuals working in alternating shift schedules, and those with cognitive impairment, as assessed by a specific screening tool—the 10-point Cognitive Screener (10-CS).

The exclusion of melatonin users was based on evidence that this substance exerts a direct influence on the circadian rhythm and may induce chronodisruption, particularly in hemodialysis patients. Experimental studies have demonstrated that the administration of melatonin to dialysis patients can restore the nocturnal rise of endogenous melatonin and improve sleep latency, fragmentation, and efficiency, highlighting its chronobiotic effect^11^. Additionally, chronobiological dysfunction is common in CKD, and melatonin plays a central role in maintaining biological rhythmicity^12^. Although other medications with sleep effects (such as sedatives and antidepressants) were considered in the analysis, they were not excluded due to their diverse and less specific mechanisms of action on chronoregulation, as well as their high in dialysis patients, which might compromise the sample’s representativeness.

The study was approved by the Ethics Committee of the Federal University of Alagoas (UFAL) (CAAE: 73105323.5.0000.5013, opinion no. 6,623,944), and all ethical procedures established by Resolutions 466/12 and 510/16 were strictly followed. Eligible participants who agreed to participate signed the Informed Consent Form (ICF).

#### Sample

The sampling method used was non-probabilistic and consecutive. Of the 481 patients initially approached, 102 refused to sign the ICF, 108 did not meet the inclusion criteria, 35 were excluded due to clinical conditions that prevented participation, 40 had incomplete responses, and 31 had cognitive impairment. Ultimately, 165 participants were included in the study.

Data collection was carried out through inter­views conducted during hemodialysis sessions in a private setting using a structured form. The form included validated questionnaires to assess excessive daytime sleepiness, sleep quality, OSA, and restless legs syndrome. Additionally, laboratory test results from the three months preceding the interview were collected.

#### Dependent Variable

The primary outcome variable of the study was major adverse cardiovascular events (MACE), defined as acute myocardial infarction (AMI), stroke, or sudden death. In this study, the composite endpoint MACE plus (MACE+) was adopted, encompassing the classic MACE events and acute events related to peripheral artery disease (PAD), thereby broadening the scope of the clinical outcome assessment^13^.

To identify these outcomes, face-to-face interviews were conducted with participants using a structured clinical form. This instrument included questions about prior cardiovascular events such as AMI and stroke, as well as hospitalizations, amputations, use of continuous positive airway pressure (CPAP), and other clinical complications. For each reported event, complementary data, including date, frequency, type, and location of occurrence, were recorded. These self-reported data were subsequently verified in medical records to validate the reported events and address potential gaps regarding dates, clinical classifications (e.g., ischemic or hemorrhagic stroke), and treatments received. In cases of discrepancies, the information in the medical records was considered. Death was classified as cardiovascular when related to cardiac or cerebrovascular causes, as recorded in the medical chart or, when necessary, confirmed by family members.

#### Independent Variables

For sample characterization and analysis of independent variables, sociodemographic and clinical information was collected through interviews and medical records, including data such as age, sex, presence of comorbidities, occurrence of complications, and cardiovascular events, as well as clinical complications related to treatment. Dialysis shift and participants’ chronotype were also recorded.

Excessive daytime sleepiness was measured using the Epworth Sleepiness Scale (ESS), a validated questionnaire that assesses the likelihood of an individual falling asleep in eight different everyday situations commonly associated with increased sleepiness. The total score ranges from 0 to 24, with scores above 10 considered indicative of excessive daytime sleepiness^14^.

Sleep quality was assessed by the Pittsburgh Sleep Quality Index (PSQI), a validated and reliable instrument that evaluates sleep quality and the presence of sleep disturbances over the preceding month. The instrument consists of 19 items generating seven components: subjective sleep quality, sleep latency, sleep duration, habitual sleep efficiency, sleep disturbances, use of sleep medication, and daytime dysfunction. The sum of these component scores results in a total score, with values above 5 indicating poor sleep quality^15,16^.

Risk of OSA was evaluated using the STOP-Bang questionnaire, a validated screening tool. This structured questionnaire consists of eight yes/no questions related to factors such as snoring, daytime fatigue, observed apneas, hypertension, body mass index (BMI), age, neck circumference, and sex. Scores range from 0 to 8, with a score ≥ 3 indicating a high risk^17,18^.

Screening for restless legs syndrome (RLS) was performed using a validated single yes-or-no question that incorporates the main diagnostic criteria: “When trying to relax or sleep at night, have you ever felt unpleasant, restless sensations in your legs that are relieved by walking or movement?”^19,20^.

The most recent laboratory tests performed up to three months prior to the interview were also considered, including hemoglobin, hematocrit, albumin, glucose, ferritin, creatinine, potassium, calcium, phosphorus, Kt/V, and parathyroid hormone.

#### Statistical Analysis

The normality of variables was assessed using the Kolmogorov-Smirnov test. For comparison of means between groups, the Student’s t-test for independent samples was used when the variable followed a normal distribution, and the Mann-Whitney test was applied when this condition was not met. For categorical variables, the chi-square test was applied.

When the expected frequency in any cell was less than five, Fisher’s exact test was used. To estimate the association between independent variables and the presence of MACE+, binary logistic regression analysis was conducted with OR and 95% confidence intervals. The significance level adopted was 5% (p < 0.05). All analyses were performed using SPSS software (version 26, Chicago, IL, USA). Additionally, to identify the main risk factors for MACE+, multivariate logistic regression models were constructed, including variables with a p-value < 0.20 in the univariate analysis to avoid exclusion bias. Variables that remained significant after adjustment were considered independent risk factors.

## Results

The final sample consisted of 165 participants, with a predominance of males (60.6%) and a mean age of 51 ± 13 years. The most frequent comorbidities were hypertension (87.9%) and diabetes mellitus (39.2%). The main clinical and laboratory parameters of the sample are presented in Table 1 and reflect the typical profile of patients with CKD undergoing hemodialysis.

**Table 1:** General characteristics of the sample (n = 165)

Variables	N (%)
Age (years)	51± 13
Sex	
Male	100 (60.6%)
Comorbidities	
Hypertension	131 (87.90%)
Diabetes Mellitus	58 (39.20%)
Ultrafiltration (L)	2.98 ± 1.01
Creatinine (mg/dL)	9.56 ± 3.03
Phosphorus (mg/dL)	4.79 ± 1.81
Hemoglobin (g/dL)	10.2 ± 1.76
Potassium (mmol/L)	5.66 ± 1.08
Kt/V	1.36 ± 0.31
Serum albumin (g/dL)	3.79 ± 0.51
Systolic blood pressure (mmHg)	151.19 ± 22.10
Diastolic blood pressure (mmHg)	82.13 ± 11.637
Body mass index (kg/m^2^)	25.60 ± 4.90
Dialysis shift	
1st shift	34 (20.60%)
2nd shift	88 (53.30%)
3rd shift	43 (26.10%)
Cardiovascular events	
Acute myocardial infarction	5 (3.40%)
Stroke	4 (2.70%)
Sudden death	11 (7.50%)

Note – Data are presented as mean ± standard deviation or absolute number (n) with percentages.

Among the clinical complications assessed, restless legs syndrome was the most frequent (29.7%) (Table 2), followed by sudden death among cardiovascular events (7.5%) (Table 1). Regarding chronotypes, the morning type predominated (67.9%). With respect to sleep quality, 73.2% of the participants reported unsatisfactory sleep, and 32.1% had with excessive daytime sleepiness. Additionally, 64.8% had an intermediate or high risk of OSA, and patients at high risk of OSA more frequently used medications for insomnia, depression, and anxiety (6 [11%] vs. 24 [25.8%], p = 0.036) (Table 2).

**Table 2:** Distribution of sleep-related variables (n = 165)

Variables	N (%)
Chronotype	
Morning	112 (67.9%)
Intermediate or afternoon	53 (32.10%)
Sleep quality	
Unsatisfactory sleep	120 (73.20%)
Excessive daytime sleepiness	53 (32.10%)
Discrepancy	50 (30.30%)
Restless legs syndrome	49 (29.70%)
OSA	
Intermediate or high OSA risk	107 (64.80%)
Sleep-Inducing Medications	
Clonazepam	15 (9.00%)
Amitriptyline	6 (3.60%)
Pregabalin	8 (4.80%)
Gabapentin	1 (0.60%)
Trazodone	1 (0.60%)
Zolpidem	2 (1.20%)
Sertraline	2 (1.20%)
Quetiapine	1 (0.60%)

Note – Data are presented as mean ± standard deviation or absolute number (n) with percentages.

When comparing the groups according to OSA risk (Table 3), individuals with intermediate or high risk of OSA were, on average, older (55.16 ± 10.08 vs. 46.74 ± 13.57 years; p < 0.001), had a higher prevalence of men(69.2% vs. 44.8%; p = 0.002), and higher body mass index (26.85 ± 4.90 vs. 23.44 ± 4.27 kg/m^2^; p < 0.001). They also had significantly greater neck circumference (40.17 ± 4.41 cm vs. 36.10 ± 3.72 cm; p < 0.001) and lower dialysis adequacy, measured by Kt/V (1.32 ± 0.31 vs. 1.44 ± 0.29; p = 0.02). In this group, the presence of RLS (23.4% vs. 10.3%; p = 0.04) and poor sleep quality (79.2% vs. 62.1%; p = 0.01) were also more frequent, suggesting a possible overlap between OSA and other sleep disorders. However, no difference was found between isolated poor sleep quality and cardiovascular outcomes.

**Table 3:** Distribution of categorical and continuous variables according to intermediate/high osa risk

Variables	No OSA Risk (n = 58)	With OSA Risk (n = 107)	p-value
Age (years)	46.74 (13.57)	55.16 (10.08)	<0.001
Men	26 (44.8%)	74 (69.20%)	0.002
Systolic Blood Pressure (mmHg)	148.77 (19.04)	152.51 (23.59)	0.27
Diabetes Mellitus	11 (20.80%)	47 (49.50%)	<0.001
Ultrafiltration (L)	2.83 (1.52)	3.05 (0.98)	0.20
PTH (pg/mL)	516.89 (490.11)	489.86 (496.54)	0.74
Serum Albumin (g/dL)	3.78 (0.50)	3.80 (0.52)	0.82
Kt/V	1.44 (0.29)	1.32 (0.31)	0.02
Potassium (mmol/L)	5.48 (1.10)	5.75 (1.06)	0.12
Hemoglobin (g/L)	10.25 (1.75)	10.16 (1.77)	0.77
Phosphorus (mg/dL)	4.79 (2.14)	4.78 (1.61)	0.97
Total Calcium (mg/dL)	9.13 (1.01)	8.85 (0.87)	0.08
Body Mass Index (kg/m^2^)	23.44 (4.27)	26.85 (4.90)	<0.001
SPI (Restless Legs Syndrome)	6 (10.30%)	25 (23.40%)	0.04
Poor Sleep Quality	36 (62.10%)	84 (79.20%)	0.01
Sleepiness	15 (25.90%)	38 (35.50%)	0.20
Hospitalization	43 (38.40%)	69 (61.60%)	0.45
CVA	53 (36.80%)	91 (63.20%)	0.63
Peripheral Artery Disease (PAD)	0 (0.00%)	1 (100.00%)	0.45
Acute Myocardial Infarction/Unstable Angina	52 (36.40%)	91 (63.60%)	0.45
MACE+	3 (5.20%)	18 (17.00%)	0.03
Sudden Death	0 (0.00%)	11 (100.00%)	0.01

Note – Data are presented as mean ± standard deviation or absolute number (n) with percentages.

When analyzing the most relevant clinical outcomes, the high-risk OSA group had a higher prevalence of diabetes mellitus (49.5% vs. 20.8%; p < 0.001), a greater frequency of MACE+ (17.0% vs. 5.2%; p = 0.03), and a higher occurrence of sudden death (11 cases vs. none; p = 0.01). Other clinical variables, such as blood pressure, hospitalization, stroke, myocardial infarction/unstable angina, excessive daytime sleepiness, and laboratory variables, showed no significant differences between the groups.

Table 4 presents the comparison of categorical and continuous variables according to the occurrence of MACE+. Participants who experienced MACE+ were significantly older compared to those without MACE+ (median age 59 years [IQR: 14.00] vs. 52 years [IQR: 18.00]; p = 0.01) and had a higher frequency of intermediate or high risk of OSA (85.7% vs. 61.5%; p = 0.03). Other variables, such as the presence of diabetes mellitus, poor sleep quality, restless legs syndrome, arterial hypertension, ultrafiltration, and BMI, showed a similar distribution between groups.

**Table 4:** Distribution of categorical and continuous variables according to the presence of mace+

Variable	Without MACE+ (n = 143)	With MACE+ (n = 21)	p-value
Age (years)	52.00 (18.00)	59.00 (14.00)	0.01
Systolic blood pressure (mmHg)	153.00 (26.50)	141.50 (35.00)	0.27
Diabetes	49 (37.40%)	9 (52.90%)	0.21
Ultrafiltration (L)	3.00 (1.00)	3.33 (0.88)	0.32
PTH (pg/mL)	321.80 (478.30)	226.00 (404.00)	0.39
Serum albumin (g/dL)	3.79 (0.51)	3.82 (0.54)	0.77
Kt/V	1.37 (0.31)	1.28 (0.32)	0.23
Potassium (mmol/L)	5.60 (1.30)	5.70 (2.20)	0.23
Hemoglobin (g/L)	10.16 (1.76)	10.53 (1.74)	0.36
Phosphorus (mg/dL)	4.51 (1.95)	4.30 (1.60)	0.52
Calcium (mg/dL)	8.94 (0.93)	8.97 (0.95)	0.90
Body Mass Index (kg/m^2^)	25.59 (5.04)	25.83 (4.37)	0.83
Unsatisfactory sleep	105 (73.90%)	15 (71.40%)	0.80
Intermediate or high risk for OSA	88 (61.50%)	18 (85.70%)	0.03
SPI (Restless Legs Syndrome)	42 (29.40%)	7 (33.30%)	0.71

Note – Data are presented as mean ± standard deviation or absolute number (n) with percentages.

Finally, in the logistic regression analysis (Table 5), intermediate or high OSA risk remained an independent risk factor for the occurrence of MACE+ (OR: 1.18; 95% CI: 1.01–1.39; p = 0.03). Other variables included in the model—such as age (OR: 1.05; 95% CI: 1.00–1.11; p = 0.07), presence of diabetes mellitus (OR: 1.33; 95% CI: 0.46–3.90; p = 0.60), and dialysis shift (OR: 2.97; 95% CI: 0.96–4.56; p = 0.11)—were not associated with MACE+.

**Table 5:** Independent risk factors for mace+ (logistic regression analysis)

Variable	OR (95% CI)	p-value
Age (years)	1.05 (1.00–1.11)	0.07
Diabetes Mellitus	1.33 (0.46–3.90)	0.60
Shift	2.97 (0.96–4.56)	0.11
Intermediate or high risk for OSA	1.18 (1.01–1.39)	0.03

Abbreviations – OR: Odds Ratio; CI: Confidence Interval (95%); OSA: Obstructive Sleep Apnea.

## Discussion

This prospective cohort study investigated the association between intermediate or high OSA risk and the occurrence of MACE+ in hemodialysis patients over a two-year follow-up period. The main findings revealed that more than 60% of participants were at intermediate or high OSA risk, and there was an independent association between MACE+ and sudden death.

Furthermore, the group at increased OSA risk had a higher proportion of individuals with diabetes, older age, higher BMI, greater neck circumference, and lower dialysis adequacy. These factors are well known to be associated with OSA and help explain the elevated cardiovascular risk in this population, either by contributing to upper airway collapse or by being linked to metabolic and inflammatory dysfunctions^21,22^. The increased neck circumference in individuals with OSA reinforces its relevance as a clinical marker of this disorder. These findings align with the study by DeLoach and Berns^23^, which demonstrated that patients with higher OSA scores had significantly elevated BMI, greater central adiposity, and poorer sleep quality and functional performance. These results suggest that increased BMI is strongly associated with sleep disorders in individuals with CKD undergoing dialysis^24^. Patients at risk for OSA also exhibited lower Kt/V values—an aspect that remains underexplored in the literature, but was reported by Masuda et al., who identified significantly lower Kt/V values in patients with sleep-related breathing disorders^25^. This relationship may reflect mechanisms such as volume overload, accumulation of uremic toxins, and chronic inflammation—all of which compromise both sleep quality and cardiovascular prognosis.

Our findings are consistent with those reported in other cohorts. In a sample of 558 incident hemodialysis patients with an average follow-up of two years, individuals with OSA were shown to have a threefold increased risk of sudden cardiac death^26^. Supporting this association, previous studies have demonstrated that nocturnal hypoxia is linked to a higher incidence of cardiovascular events and all-cause mortality among hemodialysis patients^27, 28, 29^. More recently, Prabu et al. reported from a retrospective cohort of over 800,000 hemodialysis patients that OSA was significantly associated with increased mortality risk (OR = 1.35; 95% CI: 1.32–1.37)^30^.

The findings of the present study also align with those described by Sivalingam et al., who assessed 102 patients undergoing incremental hemodialysis through home sleep studies. Approximately 44% of participants had severe OSA. Although OSA severity was not significantly associated with mortality, it was related to left ventricular hypertrophy, sustained hypertension, elevated brain natriuretic peptide (BNP), and lower Kt/V values^31^. These findings reinforce the role of OSA as a relevant contributor to structural and hemodynamic cardiovascular changes, especially when accompanied by inadequate blood pressure control and inefficient dialysis clearance^27^.

The relationship between OSA and CKD has been described as bidirectional, with reciprocal pathophysiological implications that increase morbidity and mortality in this population. On one hand, CKD contributes to the development of OSA through mechanisms such as metabolic acidosis, accumulation of uremic toxins, alterations in the sensitivity of central and peripheral chemoreceptors, and pharyngeal narrowing secondary to volume overload. On the other hand, OSA acts as an aggravating factor for CKD by promoting sustained or resistant hypertension, intermittent nocturnal hypoxia, activation of the renin-angiotensin-aldosterone system (RAAS), and persistent oxidative stress, all of which are believed to culminate in endothelial injury, tubulointerstitial fibrosis, and progressive decline in glomerular filtration rate^26,28,32^.

Among the limitations of the present study, the absence of polysomnography, which may limit the accuracy of the results, is worth highlighting. However, the STOP-BANG questionnaire has been validated in hemodialysis patients, and other studies have used the tool with similar results^33^. Socioeconomic variables such as access to treatment, income, and lifestyle habits were also not analyzed. Among study strengths, to the best of our knowledge, this is the first prospective Brazilian study to demonstrate the association between OSA risk and increased cardiovascular damage using MACE+. Our findings highlight the importance of routine OSA screening in hemodialysis patients, given that interventions such as CPAP have shown a positive impact on OSA and cardiovascular risk in both hemodialysis and peritoneal dialysis patients^33,34^.

## Conclusions

The present study revealed a high prevalence of intermediate or high OSA risk among hemodialysis patients and anassociation with the occurrence of MACE+, particularly sudden death. These findings reinforce the relevance of OSA as a potential marker forcardiovascular risk in this population and highlight the need to incorporate systematic screening strategies during dialysis follow-up.

## Data Availability

The dataset supporting the results of this study is available upon request from the corresponding author (Ellen Goes da Silva). The dataset is not publicly available due to ethical restrictions, as it contains information that could compromise participant privacy.
