# Supplementary material for: Association between obstructive sleep apnea and cardiovascular events in hemodialysis patients
Source: J Bras Nefrol. 2025 Sep 26;47(4):e20250123. doi: 10.1590/2175-8239-JBN-2025-0123en (PMC12477823; doi:10.1590/2175-8239-JBN-2025-0123en)
Supplement: Translation

# Translation: Associação entre apneia obstrutiva do sono e eventos cardiovasculares em pacientes em hemodiálise

**DOI:** 10.1590/2175-8239-JBN-2025-0123pt

**Authors:** Ellen Goes, Skarllet Cândida Silva dos Santos, Rodrigo Bezerra, Flávio Teles

**Affiliations:** 1 Universidade Federal de Alagoas, Faculdade de Medicina, Programa de Pós-Graduação em Ciências Médicas, Maceió, AL, Brasil.; 2 Universidade Federal de Alagoas, Faculdade de Medicina, Maceió, AL, Brasil.; 3 Universidade de Pernambuco, Hospital Universitário de Pernambuco, Pronto-Socorro Cardiológico Universitário de Pernambuco, Recife, PE, Brasil.; 4 Universidade Federal de Alagoas, Faculdade de Medicina, Maceió, AL, Brasil.

**Keywords:** Diálise Renal, Indicadores de Morbimortalidade, Doenças Cardiovasculares, Apneia Obstrutiva do Sono, Qualidade do Sono

## Abstract

**Introdução:**

Pacientes em hemodiálise apresentam elevado risco cardiovascular, sendo morte súbita uma das principais causas de mortalidade. Distúrbios do sono são altamente prevalentes nesse grupo, e a apneia obstrutiva do sono (AOS) tem sido associada a um pior controle da pressão arterial e a danos cardiovasculares.

**Objetivo:**

Investigar a associação entre risco intermediário ou elevado de apneia obstrutiva do sono e a ocorrência de eventos cardiovasculares maiores em pacientes em hemodiálise.

**Métodos:**

Estudo de coorte multicêntrico prospectivo realizado em três clínicas de hemodiálise entre maio de 2022 e maio de 2024. Foram incluídos 165 pacientes, entre 18 e 75 anos, em hemodiálise há pelo menos 6 meses. Avaliaram-se variáveis clínicas, laboratoriais e relacionadas ao sono, como risco de AOS (*STOP-Bang*), qualidade do sono (Pittsburgh), cronotipo (Questionário de Matutino-Vespertino) e ocorrência de eventos cardiovasculares adversos maiores (ECAM+). Os pacientes foram acompanhados por 22 a 24 meses.

**Resultados:**

Observou-se que 64,8% dos pacientes apresentaram risco intermediário ou elevado de AOS. Esse grupo demonstrou maior prevalência de diabetes e obesidade, pior qualidade do sono, mais casos de síndrome das pernas inquietas crônica e menor adequação à diálise. A ocorrência de eventos cardiovasculares maiores foi significativamente maior entre os pacientes com risco de AOS (17,0% vs. 5,2%; p = 0,03), com associação independente entre AOS e morte súbita (OR 1,18; IC95% 1,01–1,39; p = 0,03). Outros distúrbios do sono não demonstraram associação com risco cardiovascular aumentado.

**Conclusão:**

Pacientes em hemodiálise apresentaram maior risco de AOS, que demonstrou associação independente com piores desfechos cardiovasculares.

## Introdução

Pacientes com doença renal crônica (DRC) em terapia dialítica apresentam elevado risco cardiovascular, sendo a morte súbita uma das principais causas de mortalidade^1^. Os principais fatores contribuintes incluem a alta prevalência de diabetes mellitus, hipertensão arterial de difícil controle, obesidade, dislipidemia, distúrbios minerais e ósseos que levam à calcificação vascular, além de fatores como hipervolemia e hipercalemia^2,3^.

Nesse contexto, a apneia obstrutiva do sono (AOS) tem ganhado destaque como um potencial fator adicional de risco cardiovascular. A AOS está associada a piores desfechos cardiovasculares na população geral e, assim como outros distúrbios do sono, tem elevada prevalência entre indivíduos em hemodiálise^4,5^. Além disso, a AOS está relacionada ao controle inadequado da pressão arterial e é mais comum em indivíduos com diabetes e obesidade^6^. Em pacientes com DRC avançada, a AOS tem sido associada a maior ganho de peso interdialítico, pior controle da pressão arterial e menor adequação dialítica^7, 8, 9^. No entanto, ainda são escassos os estudos que investigam a correlação entre AOS e desfechos cardiovasculares em pacientes em hemodiálise^10^.

Considerando os múltiplos fatores que contribuem para o elevado risco cardiovascular dos pacientes em hemodiálise, o presente estudo teve como objetivo investigar a associação entre risco intermediário ou elevado de AOS e a ocorrência de eventos cardiovasculares adversos. Além disso, o estudo avaliou a prevalência de outros distúrbios do sono nessa população e explorou suas possíveis correlações com variáveis clínicas e cardiovasculares.

## Métodos

### Desenho do Estudo

Este estudo de coorte prospectivo envolveu três etapas de coleta de dados: maio de 2022 (inclusão dos participantes), maio de 2023 e maio de 2024. Em cada momento, foram coletadas análises clínicas e dados de hospitalização dos prontuários médicos. O estudo incluiu pacientes adultos em hemodiálise de três clínicas de nefrologia localizadas em diferentes regiões do estado de Alagoas, na região Nordeste do Brasil. A seleção de unidades geograficamente distribuídas teve como objetivo captar a diversidade sociodemográfica dos pacientes e assegurar maior representatividade da população em diálise no estado. Essa abordagem permitiu uma análise mais abrangente das variáveis investigadas, considerando as diferenças regionais nos determinantes sociais e no acesso aos serviços de saúde^5^.

Foram incluídos pacientes com idade entre 18 e 75 anos, com diagnóstico de DRC e em hemodiálise há pelo menos seis meses, com frequência regular de três sessões semanais. Os critérios de exclusão incluíram pacientes impossibilitados de responder aos questionários aplicados em decorrência das condições clínicas, aqueles com frequência irregular às sessões de hemodiálise, usuários de melatonina e indivíduos que trabalhavam em regimes de turnos alternados ou com comprometimento cognitivo, conforme avaliado por uma ferramenta de triagem específica - o *Cognitive Screener* de 10 pontos (10-CS).

A exclusão de usuários de melatonina baseou-se em evidências de que essa substância exerce influência direta no ritmo circadiano e pode induzir cronodisrupção, particularmente em pacientes em hemodiálise. Estudos experimentais demonstraram que a administração de melatonina a pacientes em diálise pode restaurar a elevação noturna da melatonina endógena e melhorar a latência, a fragmentação e a eficiência do sono, destacando seu efeito cronobiótico^11^. Além disso, a disfunção cronobiológica é comum na DRC, e a melatonina desempenha um papel central na manutenção da ritmicidade biológica^12^. Embora outros medicamentos com efeitos sobre o sono (como sedativos e antidepressivos) tenham sido considerados na análise, eles não foram excluídos em razão de seus mecanismos de ação diversos e menos específicos sobre a cronorregulação, bem como de sua elevada prevalência de uso entre pacientes em diálise, o que poderia comprometer a representatividade da amostra.

O estudo foi aprovado pelo Comitê de Ética em Pesquisa da Universidade Federal de Alagoas (UFAL) (CAAE: 73105323.5.0000.5013, parecer nº 6.623.944), e todos os procedimentos éticos estabelecidos pelas Resoluções 466/12 e 510/16 foram rigorosamente seguidos. Os participantes elegíveis participaram voluntariamente da pesquisa e assinaram o Termo de Consentimento Livre e Esclarecido (TCLE).

#### Amostra

O método de amostragem adotado foi não probabilístico e consecutivo. Dos 481 pacientes inicialmente abordados, 102 recusaram-se a assinar o TCLE, 108 não preencheram os critérios de inclusão, 35 foram excluídos devido a condições clínicas que impediram a participação, 40 apresentaram respostas incompletas e 31 apresentaram comprometimento cognitivo. Ao final, 165 participantes foram incluídos no estudo.

A coleta de dados foi realizada por meio de entrevistas conduzidas durante as sessões de hemodiálise em um ambiente privado, utilizando um formulário estruturado. O formulário incluía questionários validados para avaliação de sonolência diurna excessiva, qualidade do sono, AOS e síndrome das pernas inquietas. Adicionalmente, foram coletados os resultados de exames laboratoriais realizados nos três meses que antecederam a entrevista.

#### Variável Dependente

A variável de desfecho primário do estudo foi a ocorrência de eventos cardiovasculares adversos maiores (ECAM), definidos como infarto agudo do miocárdio (IAM), acidente vascular cerebral (AVC) ou morte súbita. Neste estudo, adotou-se o desfecho composto ECAM+, que abrange ECAMs clássicos e eventos agudos relacionados à doença arterial periférica (DAP), ampliando, assim, o escopo da avaliação de desfechos clínicos adversos^13^.

Para a identificação desses desfechos, foram realizadas entrevistas presenciais com os participantes, utilizando um formulário clínico estruturado. Esse instrumento incluiu questões sobre eventos cardiovasculares prévios, como IAM e AVC, bem como sobre hospitalizações, amputações, uso de pressão positiva contínua nas vias aéreas (CPAP) e outras complicações clínicas. Para cada evento relatado, foram registrados dados complementares, incluindo data, frequência, tipo e local da ocorrência. Esses dados autorreferidos foram posteriormente verificados nos registros médicos para validar os eventos relatados e suprir possíveis lacunas quanto a datas, classificações clínicas (por exemplo, acidente vascular cerebral isquêmico ou hemorrágico) e tratamentos recebidos. Em caso de discrepâncias, foram consideradas as informações contidas nos registros médicos. O óbito foi classificado como cardiovascular quando relacionado a causas cardíacas ou cerebrovasculares, conforme registrado no prontuário médico ou, quando necessário, confirmado pelos familiares.

#### Variáveis Independentes

Para a caracterização da amostra e análise das variáveis independentes, foram coletadas informações sociodemográficas e clínicas por meio de entrevistas e prontuários médicos, incluindo dados como idade, sexo, presença de comorbidades, ocorrência de complicações e eventos cardiovasculares, bem como complicações clínicas relacionadas ao tratamento. Também foram registrados o turno de diálise e o cronotipo dos participantes.

A sonolência diurna excessiva foi mensurada por meio da Escala de Sonolência de Epworth (ESE), um questionário validado que avalia a probabilidade de um indivíduo adormecer em oito diferentes situações do cotidiano comumente associadas ao aumento da sonolência. O escore total varia de 0 a 24, sendo que pontuações acima de 10 são consideradas indicativas de sonolência diurna excessiva^14^.

A qualidade do sono foi avaliada pelo Índice de Qualidade do Sono de Pittsburgh (PSQI), um instrumento validado e confiável que avalia a qualidade do sono e a presença de distúrbios do sono durante o mês precedente. O instrumento consiste em 19 itens que geram sete componentes do sono: qualidade subjetiva, latência, duração, eficiência habitual, distúrbios, uso de medicamentos e disfunção diurna. A soma dos escores desses componentes resulta em uma pontuação total, com valores superiores a 5 indicando má qualidade do sono^15,16^.

O risco de AOS foi avaliado utilizando o questionário *STOP-Bang*, um instrumento de triagem validado. Este questionário objetivo consiste em oito questões de resposta sim/não relacionadas a fatores como ronco, fadiga diurna, apneias observadas, hipertensão, índice de massa corporal (IMC), idade, circunferência cervical e sexo. As pontuações variam de 0 a 8, sendo que uma pontuação ≥ 3 indica risco elevado^17,18^.

A triagem para síndrome das pernas inquietas (SPI) foi realizada por meio de uma única pergunta validada, com resposta sim/não, que incorpora os principais critérios diagnósticos: “Ao tentar relaxar ou dormir à noite, você já sentiu sensações desagradáveis e inquietantes nas pernas que são aliviadas ao caminhar ou se movimentar?”^19,20^.

Também foram considerados os exames laboratoriais mais recentes, realizados até três meses antes da entrevista, incluindo hemoglobina, hematócrito, albumina, glicose, ferritina, creatinina, potássio, cálcio, fósforo, Kt/V e paratormônio.

#### Análise Estatística

A normalidade das variáveis foi avaliada utilizando o teste de Kolmogorov-Smirnov. Para a comparação das médias entre os grupos, foi utilizado o teste t de Student para amostras independentes quando a variável apresentava distribuição normal, e o teste de Mann-Whitney quando essa condição não era atendida. Para as variáveis categóricas, empregou-se o teste do qui-quadrado.

Quando a frequência esperada em qualquer célula foi inferior a cinco, utilizou-se o teste exato de Fisher. Para estimar a associação entre variáveis independentes e a presença de ECAM+, foi realizada uma análise de regressão logística binária, com cálculo do OR e intervalos de confiança de 95%. O nível de significância adotado foi de 5% (p < 0,05). Todas as análises foram realizadas utilizando o software SPSS (versão 26, Chicago, IL, EUA). Além disso, para identificar os principais fatores de risco para ECAM+, foram realizados modelos de regressão logística multivariada, incluindo variáveis com valor de p < 0,20 na análise univariada a fim de evitar viés de exclusão. As variáveis que permaneceram significativas após o ajuste foram consideradas fatores de risco independentes.

## Resultados

A amostra final consistiu em 165 participantes, com predominância do sexo masculino (60,6%) e média de idade de 51 ± 13 anos. As comorbidades mais frequentes foram hipertensão arterial (87,9%) e diabetes mellitus (39,2%). Os principais parâmetros clínicos e laboratoriais da amostra estão apresentados na Tabela 1 e refletem o perfil clássico de pacientes com DRC submetidos à hemodiálise.

**Tabela 1:** Características gerais da amostra (n = 165)

Variáveis	N (%)
Idade (anos)	51 ± 13
Sexo	
Masculino	100 (60,6%)
Comorbidades	
Hipertensão	131 (87,90%)
Diabetes Mellitus	58 (39,20%)
Ultrafiltração (L)	2,98 ± 1,01
Creatinina (mg/dL)	9,56 ± 3,03
Fósforo (mg/dL)	4,79 ± 1,81
Hemoglobina (g/L)	10,2 ± 1,76
Potássio (mmol/L)	5,66 ± 1,08
Kt/V	1,36 ± 0,31
Albumina sérica (g/dL)	3,79 ± 0,51
Pressão arterial sistólica (mmHg)	151,19 ± 22,10
Pressão arterial diastólica (mmHg)	82,13 ± 11,637
Índice de massa corporal (kg/m^2^)	25,60 ± 4,90
Turno de diálise	
1º turno	34 (20,60%)
2º turno	88 (53,30%)
3º turno	43 (26,10%)
Eventos cardiovasculares	
Infarto agudo do miocárdio	5 (3,40%)
Acidente vascular cerebral	4 (2,70%)
Morte súbita	11 (7,50%)

Nota – Os dados são apresentados como média ± desvio padrão ou número absoluto (n) com porcentagem.

Entre as complicações clínicas avaliadas, a síndrome das pernas inquietas foi a mais frequente (29,7%) (Tabela 2), seguida de morte súbita entre os eventos cardiovasculares (7,5%) (Tabela 1). Em relação aos cronotipos, predominou o tipo matutino (67,9%). No que diz respeito à qualidade do sono, 73,2% dos participantes relataram sono insatisfatório e 32,1% apresentaram sonolência diurna excessiva. Além disso, 64,8% apresentaram risco intermediário ou elevado de AOS, e os pacientes com alto risco de AOS utilizaram com maior frequência medicamentos para insônia, depressão e ansiedade (6 [11%] vs. 24 [25,8%]; p = 0,036) (Tabela 2).

**Tabela 2:** Distribuição das variáveis relacionadas ao sono (n = 165)

Variáveis	N (%)
Cronotipo	
Matutino	112 (67,9%)
Intermediário ou vespertino	53 (32,10%)
Qualidade do sono	
Sono insatisfatório	120 (73,20%)
Sonolência diurna excessiva	53 (32,10%)
Discrepância	50 (30,30%)
Síndrome das pernas inquietas	49 (29,70%)
AOS	
Risco intermediário ou elevado de AOS	107 (64,80%)
Medicamentos Indutores do Sono	
Clonazepam	15 (9,00%)
Amitriptilina	6 (3,60%)
Pregabalina	8 (4,80%)
Gabapentina	1 (0,60%)
Trazodona	1 (0,60%)
Zolpidem	2 (1,20%)
Sertralina	2 (1,20%)
Quetiapina	1 (0,60%)

Nota – Os dados são apresentados como média ± desvio padrão ou número absoluto (n) com porcentagem.

Ao comparar os grupos de acordo com o risco de AOS (Tabela 3), observou-se que os indivíduos com risco intermediário ou elevado eram, em média, mais velhos (55,16 ± 10,08 vs. 46,74 ± 13,57 anos; p < 0,001), apresentavam maior prevalência do sexo masculino (69,2% vs. 44,8%; p = 0,002) e maior índice de massa corporal (26,85 ± 4,90 vs. 23,44 ± 4,27 kg/m^2^; p < 0,001). Esses pacientes também apresentaram circunferência cervical significativamente maior (40,17 ± 4,41 cm vs. 36,10 ± 3,72 cm; p < 0,001) e menor adequação dialítica, medida pelo Kt/V (1,32 ± 0,31 vs. 1,44 ± 0,29; p = 0,02). Nesse grupo, a presença de SPI (23,4% vs. 10,3%; p = 0,04) e má qualidade do sono (79,2% vs. 62,1%; p = 0,01) também foram mais frequentes, sugerindo uma possível sobreposição entre AOS e outros distúrbios do sono. No entanto, não foi constatada diferença entre a má qualidade do sono isolada e os desfechos cardiovasculares.

**Tabela 3:** Distribuição de variáveis categóricas e contínuas de acordo com o risco intermediário/elevado de aos

Variáveis	Sem risco de AOS (n = 58)	Com risco de AOS (n = 107)	Valor de p
Idade (anos)	46,74 (13,57)	55,16 (10,08)	<0,001
Sexo Masculino	26 (44,8%)	74 (69,20%)	0,002
Pressão Arterial Sistólica (mmHg)	148,77 (19,04)	152,51 (23,59)	0,27
Diabetes Mellitus	11 (20,80%)	47 (49,50%)	<0,001
Ultrafiltração (L)	2,83 (1,52)	3,05 (0,98)	0,20
PTH (pg/mL)	516,89 (490,11)	489,86 (496,54)	0,74
Albumina Sérica (g/dL)	3,78 (0,50)	3,80 (0,52)	0,82
Kt/V	1,44 (0,29)	1,32 (0,31)	0,02
Potássio (mmol/L)	5,48 (1,10)	5,75 (1,06)	0,12
Hemoglobina (g/L)	10,25 (1,75)	10,16 (1,77)	0,77
Fósforo (mg/dL)	4,79 (2,14)	4,78 (1,61)	0,97
Cálcio Total (mg/dL)	9,13 (1,01)	8,85 (0,87)	0,08
Índice de Massa Corporal (kg/m^2^)	23,44 (4,27)	26,85 (4,90)	<0,001
SPI (Síndrome das Pernas Inquietas)	6 (10,30%)	25 (23,40%)	0,04
Baixa qualidade do sono	36 (62,10%)	84 (79,20%)	0,01
Sonolência	15 (25,90%)	38 (35,50%)	0,20
Hospitalização	43 (38,40%)	69 (61,60%)	0,45
AVC	53 (36,80%)	91 (63,20%)	0,63
Doença Arterial Periférica (DAP)	0 (0,00%)	1 (100,00%)	0,45
Infarto Agudo do Miocárdio/Angina Instável	52 (36,40%)	91 (63,60%)	0,45
ECAM+	3 (5,20%)	18 (17,00%)	0,03
Morte Súbita	0 (0,00%)	11 (100,00%)	0,01

Nota – Os dados são apresentados como média ± desvio padrão ou número absoluto (n) com porcentagem.

Ao analisar os desfechos clínicos mais relevantes, o grupo com alto risco de AOS apresentou maior prevalência de diabetes mellitus (49,5% vs. 20,8%; p < 0,001), maior frequência de ECAM+ (17,0% vs. 5,2%; p = 0,03) e maior ocorrência de morte súbita (11 casos vs. nenhum; p = 0,01). Outras variáveis clínicas, como pressão arterial, hospitalizações, acidente vascular cerebral, infarto do miocárdio/angina instável, sonolência diurna excessiva e variáveis laboratoriais, não apresentaram diferenças significativas entre os grupos.

A Tabela 4 apresenta a comparação das variáveis categóricas e contínuas de acordo com a ocorrência de ECAM+. Os participantes que apresentaram ECAM+ eram significativamente mais velhos em comparação com aqueles sem ECAM+ (idade mediana de 59 anos [IIQ: 14,00] vs. 52 anos [IIQ: 18,00]; p = 0,01) e apresentaram maior frequência de risco intermediário ou elevado de AOS (85,7% vs. 61,5%; p = 0,03). Outras variáveis, como a presença de diabetes mellitus, má qualidade do sono, síndrome das pernas inquietas, hipertensão arterial, ultrafiltração e IMC, apresentaram distribuição semelhante entre os grupos.

**Tabela 4:** Distribuição de variáveis categóricas e contínuas de acordo com a presença de ecam+

Variável	Sem ECAM+ (n = 143)	Com ECAM+ (n = 21)	Valor de p
Idade (anos)	52,00 (18,00)	59,00 (14,00)	0,01
Pressão arterial sistólica (mmHg)	153,00 (26,50)	141,50 (35,00)	0,27
Diabetes	49 (37,40%)	9 (52,90%)	0,21
Ultrafiltração (L)	3,00 (1,00)	3,33 (0,88)	0,32
PTH (pg/mL)	321,80 (478,30)	226,00 (404,00)	0,39
Albumina sérica (g/dL)	3,79 (0,51)	3,82 (0,54)	0,77
Kt/V	1,37 (0,31)	1,28 (0,32)	0,23
Potássio (mmol/L)	5,60 (1,30)	5,70 (2,20)	0,23
Hemoglobina (g/L)	10,16 (1,76)	10,53 (1,74)	0,36
Fósforo (mg/dL)	4,51 (1,95)	4,30 (1,60)	0,52
Cálcio (mg/dL)	8,94 (0,93)	8,97 (0,95)	0,90
Índice de Massa Corporal (kg/m^2^)	25,59 (5,04)	25,83 (4,37)	0,83
Sono insatisfatório	105 (73,90%)	15 (71,40%)	0,80
Risco intermediário ou elevado de AOS	88 (61,50%)	18 (85,70%)	0,03
SPI (Síndrome das Pernas Inquietas)	42 (29,40%)	7 (33,30%)	0,71

Nota – Os dados são apresentados como média ± desvio padrão ou número absoluto (n) com porcentagem.

Por fim, na análise de regressão logística (Tabela 5), o risco intermediário ou elevado de AOS manteve-se como um fator de risco independente para a ocorrência de ECAM+ (OR: 1,18; IC 95%: 1,01–1,39; p = 0,03). Outras variáveis incluídas no modelo – como idade (OR: 1,05; IC 95%: 1,00–1,11; p = 0,07), presença de diabetes mellitus (OR: 1,33; IC 95%: 0,46–3,90; p = 0,60) e turno de diálise (OR: 2,97; IC 95%: 0,96–4,56; p = 0,11) - não apresentaram associação com ECAM+.

**Tabela 5:** Fatores de risco independentes para ecam+ (análise de regressão logística)

Variável	OR (IC95%)	Valor de p
Idade (anos)	1,05 (1,00–1,11)	0,07
Diabetes Mellitus	1,33 (0,46–3,90)	0,60
Turno	2,97 (0,96–4,56)	0,11
Risco intermediário ou elevado de AOS	1,18 (1,01–1,39)	0,03

Abreviaturas – OR: *Odds Ratio* (razão de chances); IC: Intervalo de Confiança (95%); AOS: Apneia Obstrutiva do Sono.

## Discussão

Este estudo de coorte prospectivo investigou a associação entre risco intermediário ou elevado de AOS e a ocorrência de ECAM+ em pacientes em hemodiálise durante um período de acompanhamento de dois anos. Os principais achados revelaram que mais de 60% dos participantes apresentaram risco intermediário ou elevado de AOS, e houve uma associação independente entre ECAM+ e morte súbita.

Além disso, o grupo com risco aumentado de AOS apresentou uma proporção maior de indivíduos com diabetes, idade avançada, IMC elevado, maior circunferência cervical e menor adequação dialítica. Esses fatores são bem conhecidos por estarem associados à AOS e ajudam a explicar o risco cardiovascular elevado nessa população, seja por contribuírem para o colapso das vias aéreas superiores, seja por estarem ligados a disfunções metabólicas e inflamatórias^21,22^. O aumento da circunferência cervical em indivíduos com AOS reforça sua relevância como marcador clínico desse distúrbio. Esses achados estão em consonância com o estudo de DeLoach and Berns^23^, que demonstrou que pacientes com escores mais elevados de AOS apresentavam IMC significativamente elevado, maior adiposidade central e pior qualidade do sono e desempenho funcional. Esses resultados sugerem que o aumento do IMC está fortemente associado a distúrbios do sono em indivíduos com DRC em diálise^24^. Pacientes em risco de AOS também apresentaram valores mais baixos de Kt/V – um aspecto que permanece pouco explorado na literatura, mas é corroborado por Masuda et al., que identificaram valores significativamente mais baixos de Kt/V em pacientes com distúrbios respiratórios relacionados ao sono^25^. Essa relação pode refletir mecanismos como sobrecarga volêmica, acúmulo de toxinas urêmicas e inflamação crônica – todos fatores que comprometem tanto a qualidade do sono quanto o prognóstico cardiovascular.

Nossos achados são consistentes com os relatados em outras coortes. Em uma amostra de 558 pacientes incidentes em hemodiálise, com um acompanhamento médio de dois anos, indivíduos com AOS demonstraram ter um risco três vezes maior de morte cardíaca súbita^26^. Corroborando essa associação, estudos anteriores haviam demonstrado que a hipóxia noturna está associada a uma maior incidência de eventos cardiovasculares e mortalidade por todas as causas entre pacientes em hemodiálise^27, 28, 29^. Mais recentemente, Prabu et al. demonstraram, em uma coorte retrospectiva de mais de 800.000 pacientes em hemodiálise, que a AOS esteve significativamente associada ao risco aumentado de mortalidade (OR = 1,35; IC 95%: 1,32–1,37)^30^.

Os achados do presente estudo também estão alinhados com os descritos por Sivalingam et al., que avaliaram 102 pacientes submetidos à hemodiálise incremental por meio de estudos domiciliares do sono. Aproximadamente 44% dos participantes apresentaram AOS grave. Embora a gravidade da AOS não tenha se associado significativamente à mortalidade, ela esteve relacionada à hipertrofia ventricular esquerda, hipertensão sustentada, peptídeo natriurético cerebral (BNP) elevado e valores mais baixos de Kt/V^31^. Esses achados reforçam o papel da AOS como um contribuinte relevante para alterações cardiovasculares estruturais e hemodinâmicas, especialmente quando acompanhada de controle inadequado da pressão arterial e depuração ineficiente da diálise^27^.

A relação entre AOS e DRC tem sido descrita como bidirecional, com implicações fisiopatológicas recíprocas que aumentam a morbidade e a mortalidade nessa população. Por um lado, a DRC contribui para o desenvolvimento da AOS por meio de mecanismos como acidose metabólica, acúmulo de toxinas urêmicas, alterações na sensibilidade dos quimiorreceptores centrais e periféricos e estreitamento da faringe secundário à sobrecarga volêmica. Por outro lado, a AOS atua como um fator agravante para a DRC, ao promover hipertensão sustentada ou resistente, hipóxia noturna intermitente, ativação do sistema renina-angiotensina-aldosterona (SRAA) e estresse oxidativo persistente, todos fatores que se acredita culminarem em lesão endotelial, fibrose tubulointersticial e declínio progressivo da taxa de filtração glomerular^26,28,32^.

Entre as limitações do presente estudo, destaca-se a ausência de polissonografia, o que pode limitar a precisão dos resultados. No entanto, o questionário STOP-BANG foi validado em pacientes em hemodiálise, e outros estudos utilizaram a ferramenta com resultados semelhantes^33^. Variáveis socioeconômicas, como acesso a tratamento, renda e estilo de vida, também não foram analisadas. Entre os pontos fortes do estudo, até onde é de nosso conhecimento, este é o primeiro estudo prospectivo brasileiro a demonstrar a associação entre o risco de AOS e o aumento dos danos cardiovasculares utilizando ECAM+. Nossos achados destacam a importância da triagem de rotina para AOS em pacientes em hemodiálise, uma vez que intervenções como o uso de CPAP demonstraram um impacto positivo na AOS e no risco cardiovascular tanto em pacientes em hemodiálise quanto em diálise peritoneal^33,34^.

## Conclusões

O presente estudo revelou uma alta prevalência de risco intermediário ou elevado de AOS entre pacientes em hemodiálise e sua associação com a ocorrência de ECAM+, particularmente morte súbita. Esses achados reforçam a relevância da AOS como um potencial marcador de risco cardiovascular nessa população e ressaltam a necessidade de incorporar estratégias de triagem sistemática durante o acompanhamento da diálise.

## Data Availability

Todo o conjunto de dados que dá suporte aos resultados deste estudo está disponível mediante solicitação à autora correspondente (Ellen Goes da Silva). O conjunto de dados não está publicamente disponível devido a restrições éticas, pois contém informações que podem comprometer a privacidade dos participantes da pesquisa.
